# Transcriptomic analysis to elucidate the effects of high stocking density on grass carp (*Ctenopharyngodon idella*)

**DOI:** 10.1186/s12864-021-07924-4

**Published:** 2021-08-16

**Authors:** Yan He, Hongyan Yu, Honggang Zhao, Hua Zhu, Qingjing Zhang, Anqi Wang, Yubang Shen, Xiaoyan Xu, Jiale Li

**Affiliations:** 1 Key Laboratory of Freshwater Aquatic Genetic Resources Ministry of Agriculture and Rural Affairs, Shanghai Ocean University, Shanghai, China; 2 National Demonstration Center for Experimental Fisheries Science Education, Shanghai Ocean University, Shanghai, China; 3 Shanghai Engineering Research Center of Aquaculture, Shanghai Ocean University, Shanghai, China; 4 Department of Natural Resources, Cornell University, 14853 Ithaca, New York USA; 5 Beijing Key Laboratory of Fishery Biotechnology, Beijing Fisheries Research Institute, 100068 Beijing, China

**Keywords:** *Ctenopharyngodon idella*, High stocking density, Transcriptome, Immune function, Metabolism

## Abstract

**Background:**

Grass carp (*Ctenopharyngodon idella*) is one of the most widely cultivated fishes in China. High stocking density can reportedly affect fish growth and immunity. Herein we performed PacBio long-read single-molecule real-time (SMRT) sequencing and Illumina RNA sequencing to evaluate the effects of high stocking density on grass carp transcriptome.

**Results:**

SMRT sequencing led to the identification of 33,773 genes (14,946 known and 18,827 new genes). From the structure analysis, 8,009 genes were detected with alternative splicing events, 10,219 genes showed alternative polyadenylation sites and 15,521 long noncoding RNAs. Further, 1,235, 962, and 213 differentially expressed genes (DEGs) were identified in the intestine, muscle, and brain tissues, respectively. We performed functional enrichment analyses of DEGs, and they were identified to be significantly enriched in nutrient metabolism and immune function. The expression levels of several genes encoding apolipoproteins and activities of enzymes involved in carbohydrate enzymolysis were found to be upregulated in the high stocking density group, indicating that lipid metabolism and carbohydrate decomposition were accelerated. Besides, four isoforms of grass carp major histocompatibility complex class II antigen alpha and beta chains in the aforementioned three tissue was showed at least a 4-fold decrease.

**Conclusions:**

The results suggesting that fish farmed at high stocking densities face issues associated with the metabolism and immune system. To conclude, our results emphasize the importance of maintaining reasonable density in grass carp aquaculture.

**Supplementary Information:**

The online version contains supplementary material available at 10.1186/s12864-021-07924-4.

## Introduction

Grass carp (*Ctenopharyngodon idella*) is a fish species with the largest reported production (> 6 million tons per year) in aquaculture globally [1]. Grass carp culture mainly involves semi-intensive and intensive culture [2]. To maximize the efficiency of aquaculture systems, high-density intensive culture is an inevitable choice [3]. Stocking density and disease management are pivotal factors in fish farming to enhance productivity and profitability [4]. An increased demand of aquaculture products results in high stocking density, ultimately resulting in a decline in the overall health and welfare of farmed fish. Several studies have been conducted to evaluate the effects of rearing density on variables such as fish growth and survival, food intake, and hormonal changes. In a recent study, it was reported that high stocking density is associated with alterations in fish behavior, metabolism, and immune function [5].

Next-generation sequencing can be broadly classified into short- and long-read sequencing. The combination of long- and short-read sequencing has been applied to detect low-expression isoforms and elucidate functional gene dynamics [6]. The identification of differentially expressed genes (DEGs) can provide insights into the stress response triggered upon encountering high stocking density environments and facilitate the development of molecular markers for breeding. Stress-related molecules have been studied using Illumina sequencing. PacBio SMRT sequencing can provide information on transcript diversity, including alternative splicing (AS), alternative polyadenylation (APA), and long noncoding RNAs (lncRNAs) [7]. AS can generate various mRNAs and significantly enhance regulatory capacities and proteomic complexities [8]. APA acts in association with AS to regulate gene expression. It plays a key role in translation efficiency, stability, and localization of mRNA and alters protein-coding sequences [9]. lncRNA can participate in a variety of biological functions, including cis or trans transcription regulation, nuclear domain organization, and protein or RNA molecule regulation [10].

Studies on fish stocking densities have been conducted in many fish species, including grass carp [11], large yellow croaker [12], Amur sturgeon [13], and Nile tilapia [4]. Crowding stress or high stocking density has been reported to induce complement *C1r* and *C1q* in large yellow croaker, increase *CYP 1 A* expression levels in Amur sturgeon, and upregulate lipid and nitrogen transport-related genes in Nile tilapia.

In the present study, we used PacBio long-read single-molecule real-time (SMRT) sequencing to obtain the full-length transcriptome of grass carp; further, our aim was to detect AS variants, APA sites, and lncRNAs. Illumina RNA-seq was used to polish SMRT transcripts and identify DEGs in the brain, intestine, and muscle samples obtained from fish exposed to different stocking densities. We believe that our results can serve as a reference database for isoform analysis in grass carp. Further, the identified DEGs should help improve our understanding of the detailed physiological mechanism used by *C. idella* to deal with high stocking density stress.

## Results

### PacBio SMRT Sequencing Data Analysis

The PacBio Sequel platform was used to capture full-length sequences. In total, 17,422,835 subreads (31.04 Gb) were obtained, with an average read length of 1,782 bp and N50 of 2,583 bp (Table 1). At the sequence detection step, 391,501 CCS reads were captured containing the poly-A tail and 5′- and 3′-primers. Further, 385,367 full-length non-chimeric reads were identified with low artificial concatemers, and the mean length was 2,733 bp (Table 1). The full-length non-chimeric reads were corrected with the Illumina short reads. Overall, 220,474 Illumina short reads were obtained, and the mean length was 2,906 bp and N50 was 3,867 bp (Table 1). After removing redundant sequences, 79,148 high-quality transcripts were retained, and the mean length was 2,612 bp and N50 was 3,261 bp. In total, 185,869 reads (84.3 %) were successfully mapped to the grass carp genome, with 1,999 (2.5 %) sequences being identical to the reference, 53,448 (67.5 %) identified as novel isoforms for 14,946 known genes, and 23,701 (29.9 %) identified as novel isoforms for 18,827 novel genes (Table 1).

**Table 1:** Summary of SMRT-Seq dataset

Category	Dataset
Subreads base (G)	31.04
Subreads number	17,422,835
Average subreads length	1,782
Subreads N50	2,583
CCS	462,629
5’-primer	436,445
3’-primer	436,247
Poly-A	415,941
Full length	391,501
FLNC	385,367
Average FLNC read length	2,733
Polished consensus reads	220,474
Average consensus reads length	2,906
Polished N50	3,867
Total reads	220,474
Mapped polished consensus reads	185,869(84.3 %)
High-quality isoforms number	79,148
Isoform mean length	2,612
Isoform N50	3,261
Isoforms of known genes	1,999(2.5 %)
Novel isoform of known genes	53,448(67.5 %)
Isoforms of novel genes	23,701(29.9 %)
New genes number	18,827

### Improving Grass Carp Genome Annotation Using PacBio SMRT Sequencing Data

**Fig. 1:**
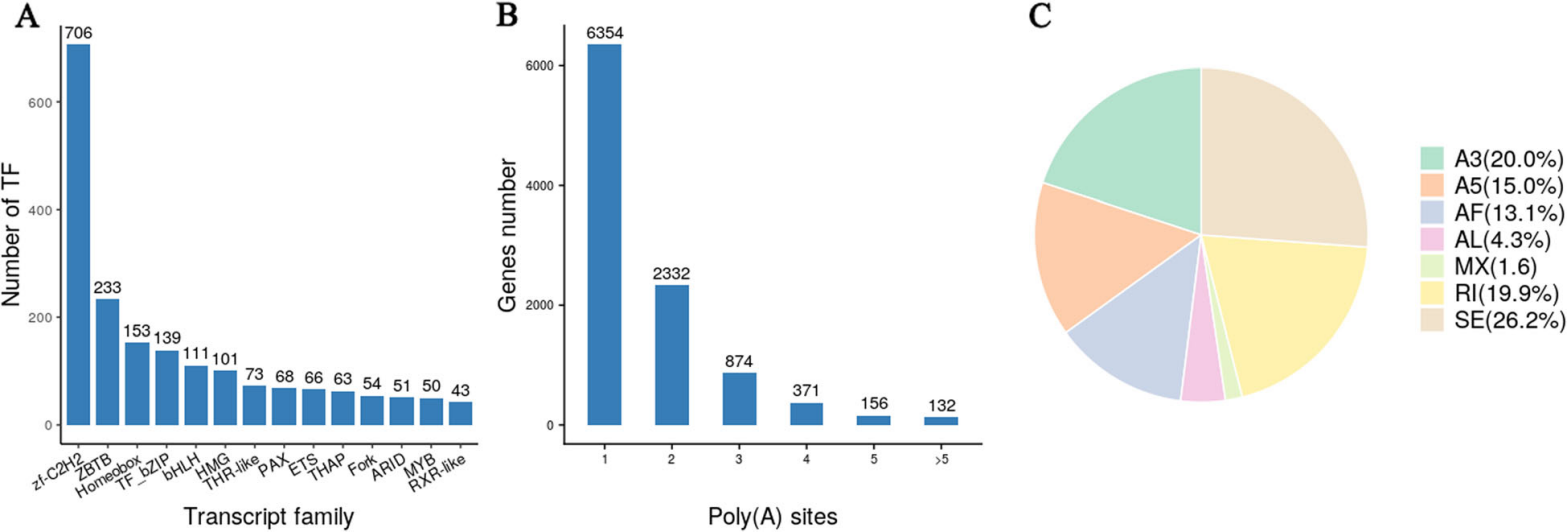
TF, AS, and APA analyses of SMRT-seq. (**A**) Distribution of the number of TF. (**B**) Distribution of the number of APA sites per gene. (**C**) The statistics of alternative splicing events. SE: skipped exon; MX: mutually exclusive exon; A5: alternative 5’ splice site; A3: alternative 3’ splice site; RI: retained intron; AF: alternative first exon; AL: alternative last exon. This figure was drawn with ggplot2 version 3.3.3 [14] and cowplot version 1.1.1 [15]

We detected a total of 79,148 transcripts and 33,773 genes, of which 14,946 were known genes and 18,827 were novel genes. Further, 79.2 % (240 genes) were complete single-copy BUSCOs, 47.5 % (144 genes) were complete duplicated BUSCOs, 31.7 % (96 genes) were fragmented BUSCOs, and 16.5 % (50 genes) were entirely missing BUSCOs. Coding region sequences and their corresponding amino acid sequences were analyzed using TransDecoder v3.0.0. In this study, the positions of 16,691 genes in the genome were optimized (Table S1), leading to the detection of 13,607 gene loci; 3,084 gene loci were new. In total, 2,495 fusion genes were identified in the PacBio library and were validated using transcriptome datasets (Table S1). Based on the AnimalTFDB 2.0 database, 2,421 transcripts were predicted to be transcription factors. The main transcription factors identified belonged to the zf-C2H2, ZBTB, Homeobox, bZIP, and bHLH families (Fig. 1 A).

### APA and AS Analyses

To identify differential polyadenylation sites (PASs), we investigated the 3′-ends of transcripts from Iso-SEq. Overall, 10,219 genes had at least one PAS, and 288 genes had at least five PAS (Fig. 1B; Table S2). The highest number of PASs was 24, which were associated with ceruloplasmin. Further, 8,009 genes, including 7,897 know and 112 novel genes, showed 22,013 AS events (Table S3). In addition, 4,582 genes with AS events had ≥ 2 isoforms. The commonest types of AS events were skipped exon (26.17 % events), alternative 3′-splice site (19.96 % events), and retained intron (19.86 % events) (Fig. 1 C). The ratio of AS events to genes was 2.75. The highest number of isoforms (69) was found for calpastatin.

### lncRNA Prediction

**Fig. 2:**
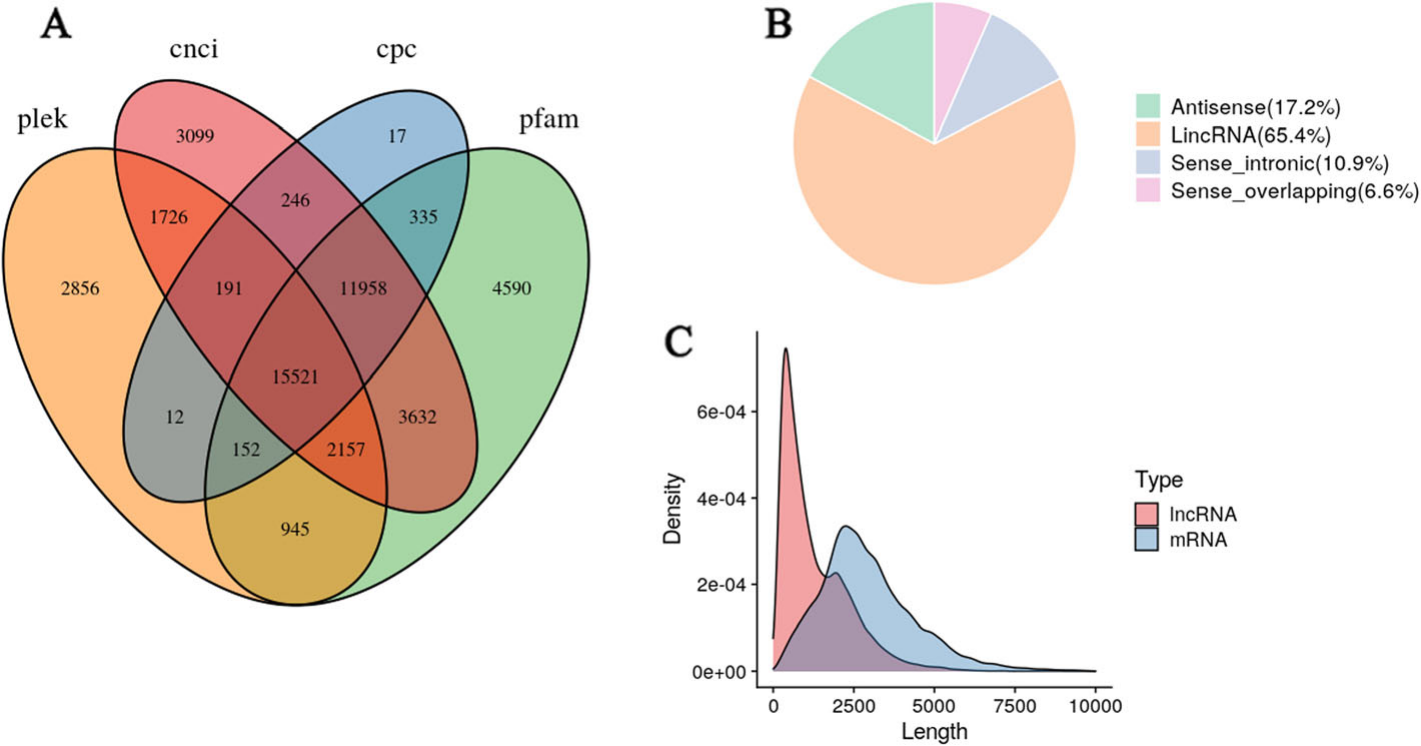
LncRNA analysis of SMRT-seq. (**A**) Venn diagram showing the number of lncRNAs annotated by CPC, CNCI, CPAT, and Pfam. (**B**) Distribution of four types of lncRNA. (**C**) Numbers of exons per lncRNA or per mRNA. This figure was drawn with VennDiagram version 1.6.20 [16], ggplot2 version 3.3.3 [14] and cowplot version 1.1.1 [15]

A total of 15,521 lncRNAs (mean length = 1,292 bp) were identified, and were shorter than the mRNA (Fig. 2 A and 2 C). LncRNA were in the range of 1 to 15 in exon number, with an average of 1.13, and 14,049 (90.52 %) lncRNAs were detected with a single exon (Table S4). In the range of 9.9 to 73.96 in guanine-cytosine (GC) content, with an average of 35.35. All lncRNAs were classified into four groups: 10,148 (65.38 %) long intervening noncoding RNAs, 2,667 (17.18 %) antisense lncRNAs, 1,684 (10.85 %) sense intronic lncRNAs, and 1,022 (6.58 %) sense overlapping lncRNAs (Fig. 2B).

### DEG Analyses

Overall, 2,321 genes were regulated in this study in all tissues, and 1,032 AS events, 1,407 APA sites, and 654 lncRNAs were found in all DEGs (Table S5). Among them 1,300 DEGs were distributed in the intestine (660 up- and 640 down-regulated), 1,004 in the muscle (296 up- and 708 down-regulated), and 228 in the brain (69 up- and 159 down-regulated) (Table S5). In addition, 572 AS events, 784 APA sites, and 374 lncRNAs were found in 1,300 intestine DEGs; 468 AS events, 655 APA sites, and 301 lncRNAs were found in 1,004 muscle DEGs; and 95 AS events, 125 APA sites, and 75 lncRNAs were found in 228 brain DEGs (Table S5).

**Fig. 3:**
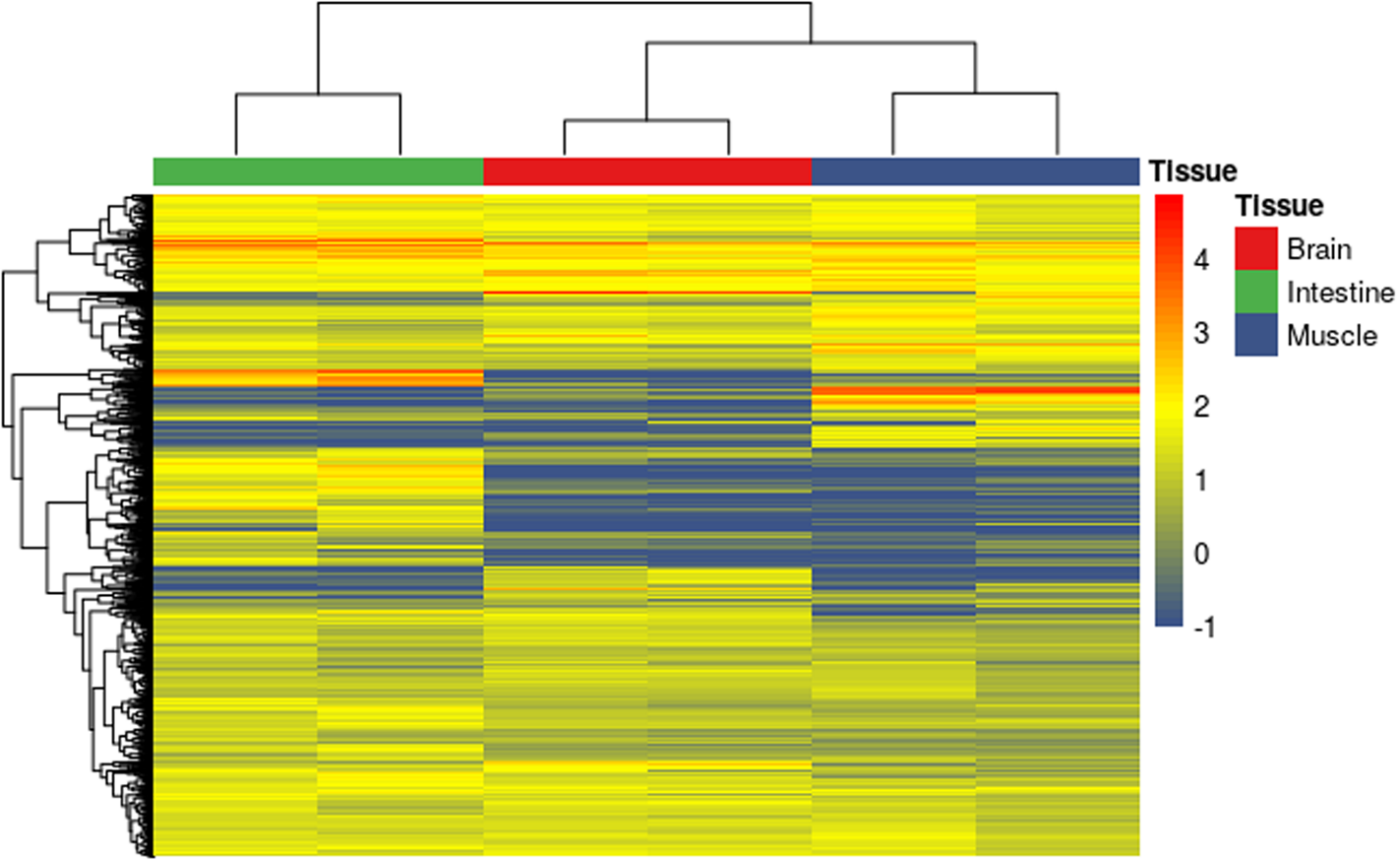
Heat map of DEGs identified from different density groups. This figure was drawn with pheatmap version 1.0.12 [17]

**Fig. 4:**
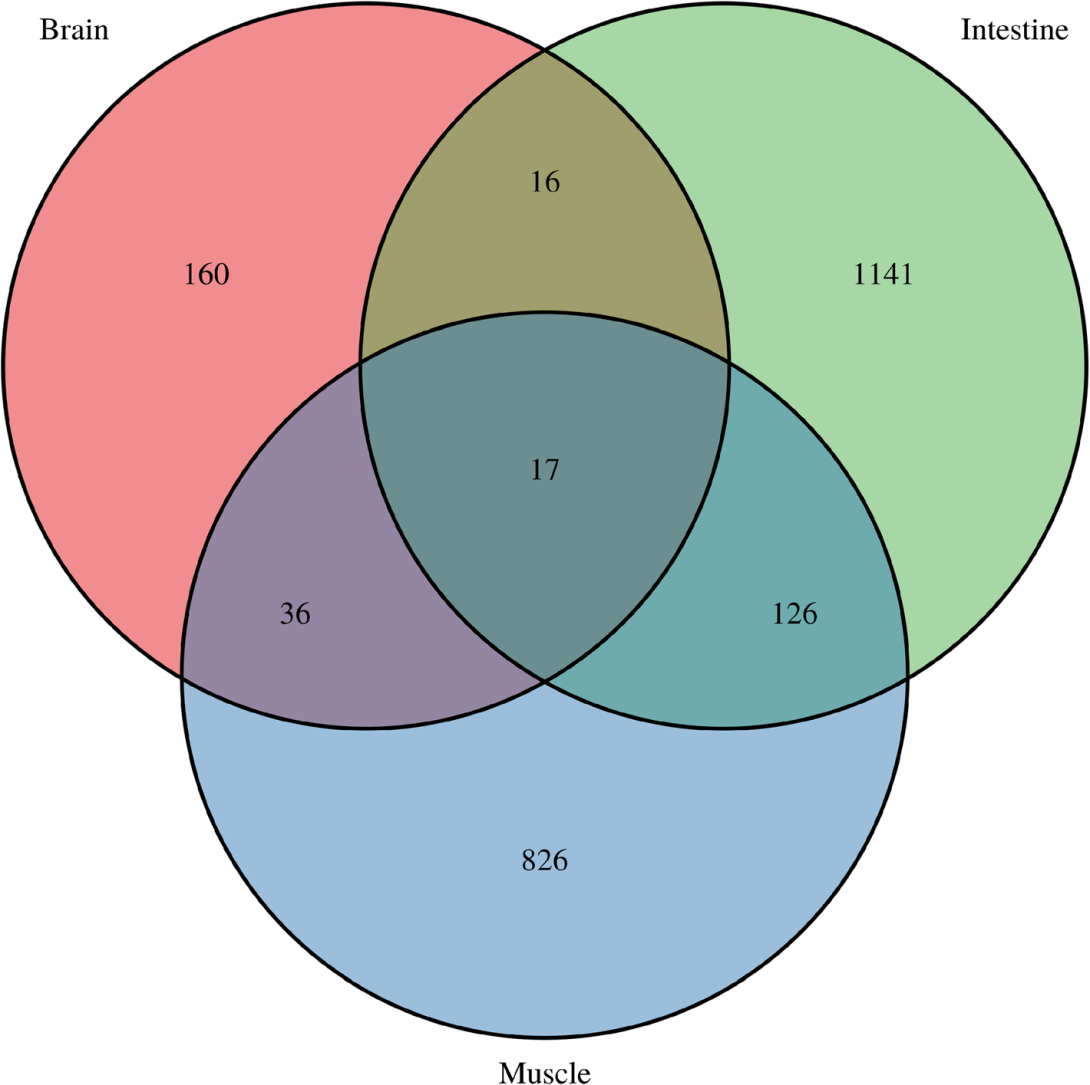
Venn diagram of DEGs from three tissues. This figure was drawn with VennDiagram version 1.6.20 [16]

The heatmap clustering analysis indicated that the expression pattern of DEGs in the brain and muscle was similar (Fig. 3). We found that 17 genes (1 co-up-regulated, 13 co-down-regulated, and 3 showing a negative relationship) were simultaneously dysregulated in all tissues (Fig. 4, Table S6); the majority of these were involved in lipid metabolism, immunoregulation, glycometabolism, and cell cycle.

**Fig. 5:**
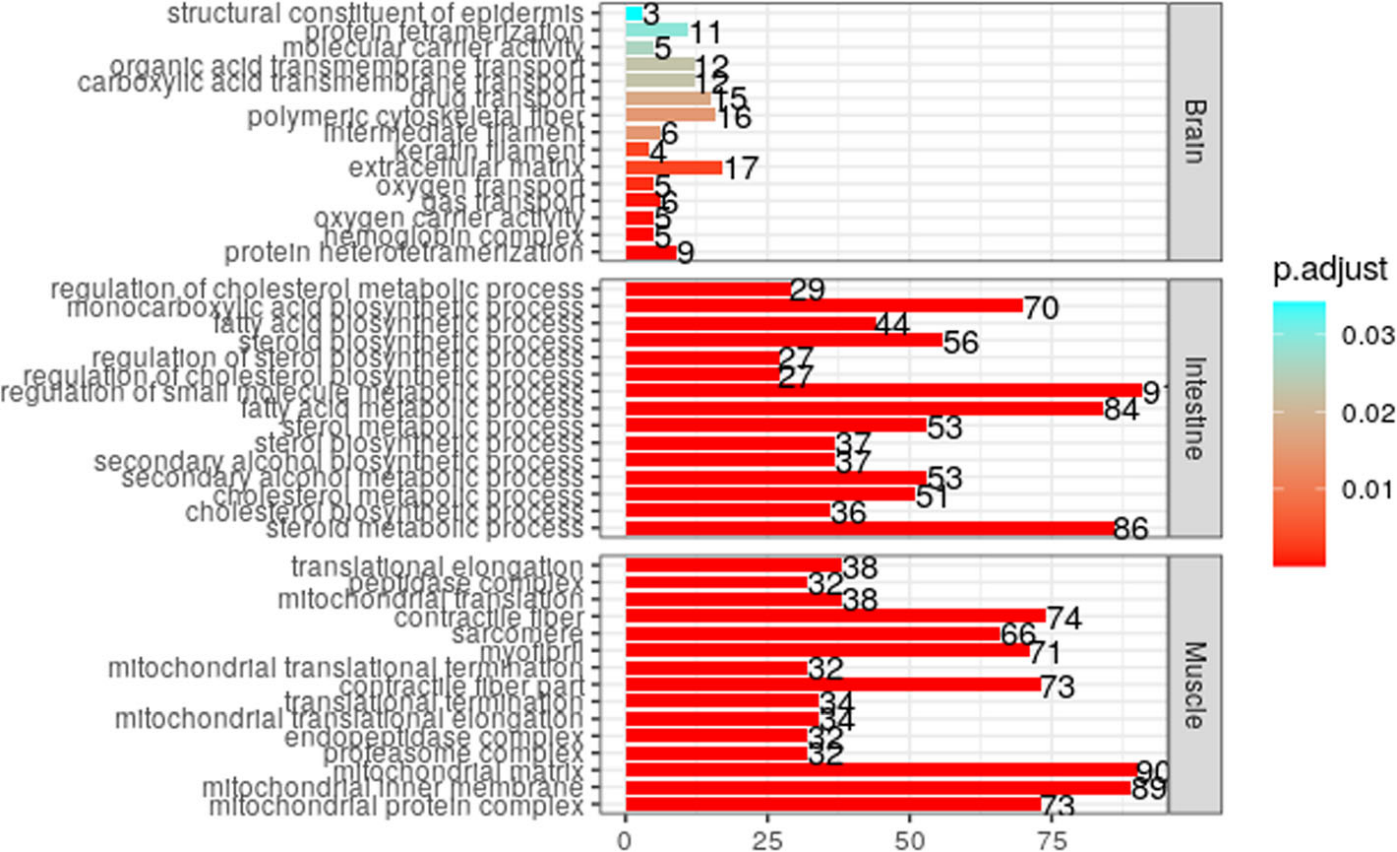
Top 25 GO terms enriched in brain, intestine and muscle DEGs. This figure was drawn with ggplot2 version 3.3.3 [14]

GO term enrichment analyses showed that DEGs in the intestine, muscle, and brain were associated with 411, 370, and 21 terms, respectively (Table S7). In the intestine, DEGs were enriched in steroid metabolic process, cholesterol biosynthetic process, and fatty acid metabolic process (Fig. 5). In the muscle, the most common enriched terms were mitochondrial protein complex, mitochondrial inner membrane, and mitochondrial matrix (Fig. 5). In the brain, DEGs were enriched in protein heterotetramerization, hemoglobin complex, and oxygen carrier activity (Fig. 5).

**Fig. 6:**
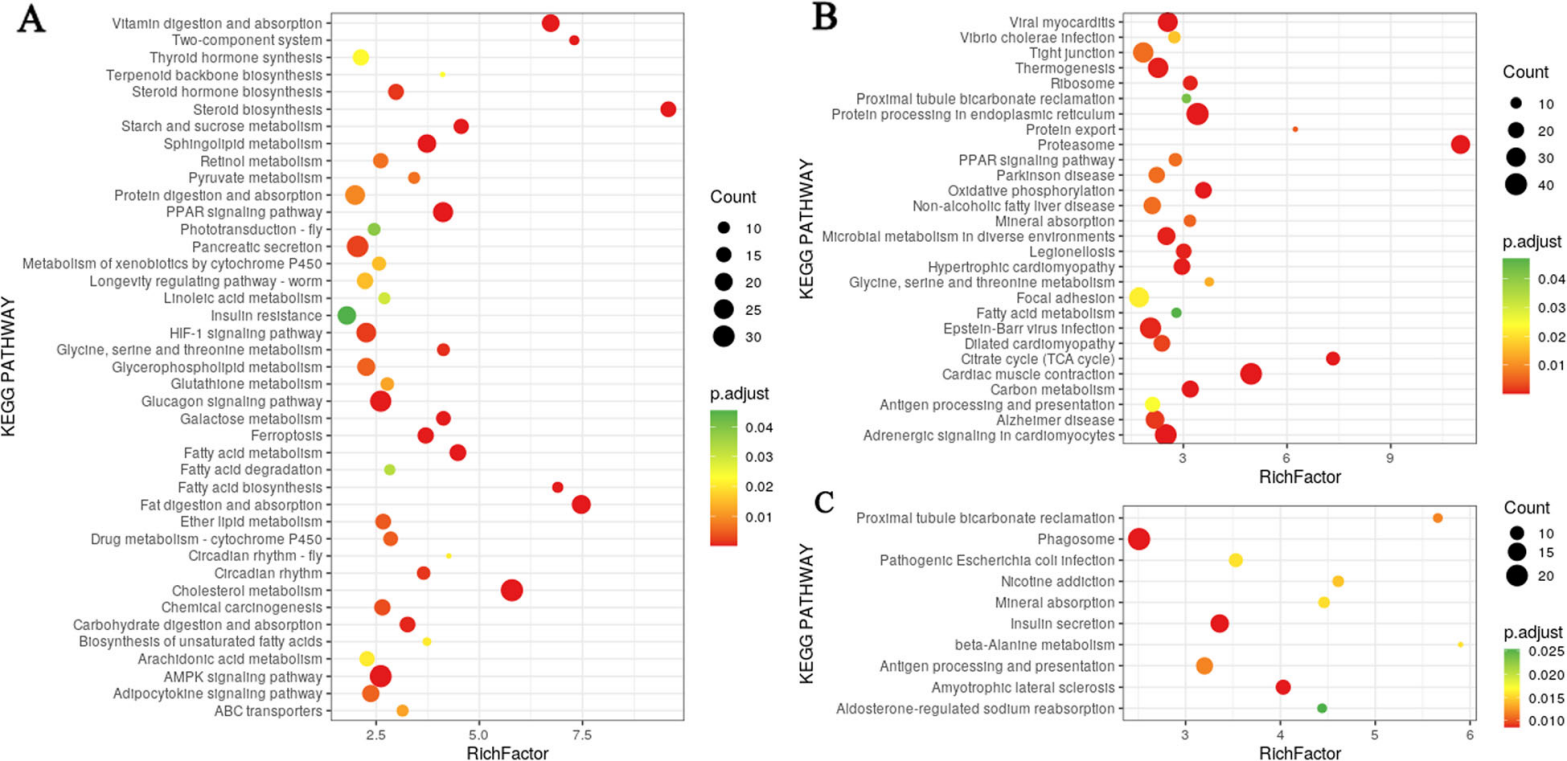
KEGG pathways enriched in (**A**) intestine, (**B**) muscle and (**C**) brain DEGs. This figure was drawn with ggplot2 version 3.3.3 [14]

Further, KEGG pathway analyses revealed that annotated DEGs were significantly enriched in 73 pathways (Fig. 6, Table S8). DEGs in the intestine were highly clustered in lipid metabolism, such as cholesterol metabolism, fat digestion and absorption, and steroid biosynthesis, and in the muscle and brain, DEGs were highly clustered in antigen processing and presentation (Table S8). To verify our Illumina sequencing data, 12 DEGs (four for each tissue) were randomly selected for qRT-PCR analysis (Figure S1). The expression patterns identified using qRT-PCR for all tested genes were in agreement with our Illumina RNA-seq data.

## Discussion

Fish reared at different stocking densities have been observed to show issues pertaining to growth performance and feed conversion [18]. In high density environments, fish tend to have negative experiences, such as unintended interaction with each other, struggle for food, and increased handling. These stress factors may cause hormone dysregulation, lower reproduction, and immunodeficiency [19, 20], in addition to alterations in digestive mechanisms, utilization of energy, and secretion of neurotransmitters [21]. Herein to gain insights into the complexity and diversity of the grass carp transcriptome, we extracted RNA from nine tissues (the muscle, gill, kidney, heart, spleen, intestine, liver, fin, and brain) and mixed them in equivalent quantities. To better understand the effect of stocking density on grass carp, we used the muscle, intestine, and brain tissues for PacBio SMRT sequencing and Illumina RNA-sEq. After transcriptome analysis, some candidate genes were identified to putatively play a key role.

SMRT sequencing generates longer reads and thus has the advantage over short-read sequencing for gene structure analyses [22]. The total AS events were 22,013 with the event/gene ratio being 2.75, which was higher than that reported for fugu (2.21) and zebrafish (1.74) [23]. Further, we found that complement C4b showed the most AS events among all annotated genes. The large number of AS events (69) in grass carp complement C4b implies the presence of multiple isoforms and its enormous potential in immunomodulation, warranting further investigations.

We found that 30.3 % grass carp genes showed APA sites. In zebrafish, 55 % of adult *Danio rerio* genes were found to APA sites [24]. This difference can be attributed to 3′-UTR prolongation during embryonic development, which could result in a higher number of PASs [25]. Among all annotated genes, the gene encoding ceruloplasmin showed the maximum number of PASs (24 sites), much higher than that encoding protein quaking-A (14 sites). Multiple isoforms of the human ceruloplasmin gene have been described, showing varying number of PASs and AS events and performing distinct functions under diverse physiological conditions [26]. Herein the gene encoding ceruloplasmin in grass carp showed 24 APA sites and 52 AS isoforms, suggesting that ceruloplasmin performs various functions depending on the environment. Further, 90.5 % lncRNAs showed a single exon, with the average exon number being 1.1, which was much lower than that for mRNAs (7.1), indicating that grass carp lncRNAs are less complex than mRNAs. Most lncRNAs (65.4 %) were classified as long intervening noncoding RNA, consistent with the results reported for zebrafish and mammals [27–29]. In this study, genes with APA sites and AS events accounted for > 50 % and > 40 % of all DEGs, respectively, and lncRNAs made up approximately 30 % of all DEGs. As is already known, AS and APA are important mechanisms for gene regulation, generating distinct mRNA isoforms from the same gene [30, 31]. lncRNAs are involved in the regulation of transcriptional activation, RNA processing, and mRNA translation [32]. A high proportion of AS events, APA sites, and lncRNAs thus indicates their potential to influence gene expression under different stocking densities.

Upon functional annotation, 14 DEGs were identified to be commonly dysregulated, and these were mainly related to lipid metabolism, glycometabolism, immunology, and cellular homeostasis. Among these 14 DEGs, the expression levels of the genes encoding angiopoietin-like 4 (*angptl4*), insulin receptor substrate 2 (*irs2*), pyruvate dehydrogenase kinase 4 (*pdk4*), thioredoxin-interacting protein (*txnip*), and MHC class II antigen chains were downregulated, and those of the gene encoding period circadian protein homolog 2 (*per2*) were upregulated in the muscle, intestine, and brain.

ANGPTL4 is an inhibitor of lipoprotein lipase, which can inhibit triglyceride clearance and increase plasma triglyceride levels [33]. IRS2, a cytoplasmic protein, is a key target of the insulin receptor tyrosine kinase and facilitates the response to insulin [34]. The downregulation of *angptl4* and *irs2* expression levels is suggestive of lower triglyceride and fat content in the HD group [35]. Furthermore, in the intestine, DEGs were significantly enriched in lipid anabolism, fat digestion and absorption, cholesterol metabolism, and steroid biosynthesis. In the intestine, the expression levels of the genes encoding lipoproteins, such as APOA1, APOB, APOC1, and APOE, were upregulated in the HD group (Table S9), potentially leading to accelerated lipid transportation [36–38]. Moreover, the upregulation of ATP-binding cassette transporter genes in the intestine may induce cholesterol efflux from cells and inhibit tissular lipid accumulation [39, 40] (Table S9). The upregulation of lipid metabolism-related genes may lead to fat reduction in fish, which explains why fish reared in high stocking densities usually show lower weight. Besides, *per2*, a core clock gene, is evidently induced in humans during weight loss [41]. The upregulation of grass carp *per2* in the aforementioned three tissues is likely due to fish emaciation under high stocking density environments.

PDK4 downregulation causes carbohydrate catabolism to switch from glycolysis to oxidative phosphorylation and promotes the complete decomposition of glucose [42]. In glycometabolism, the absence of TXNIP leads to the muscle and adipose tissues absorbing excess glucose, consequently decreasing circulating glucose levels [43–45]. The downregulation of *pdk2* and *txnip* expression levels thus indicates that blood glucose levels are reduced in grass carp. Furthermore, the activities of various enzymes (lactase, maltase-glucoamylase, hexokinase 4, and cytosolic beta-glucosidase) involved in carbohydrate enzymolysis [46–49] were increased. We also found that GLUT5 was upregulated (Table S10); GLUT5 is an key member of the glucose transporter protein family and is known to facilitate fructose uptake by cells [50]. To summarize, the expression profile of carbohydrate metabolism-related genes indicated that carbohydrate decomposition and glucose uptake by cells were enhanced, leading to low blood glucose levels in the HD group.

The main function of MHC class II molecules is to present antigens to naive CD4 + T cells [51]. The absence of MHC class II expression results can result in a severe primary immunodeficiency disease in humans, which can prove to be fatal [52]. In this study, grass carp MHC class II antigen alpha and beta chains in the three tested tissues showed at least a 4-fold decrease. This suggests that fish reared at high stocking densities experience immune dysfunction. Interestingly, according to the GO enrichment analysis of DEGs, MHC protein binding, MHC class II protein binding, and MHC class II receptor activity were enriched both in the brain and muscle. This implies that high stocking density disturbed the antigen presentation to CD8 + cells by MHC I molecules in the brain and muscle tissues. Thus, it appears that fish farmed at high stocking densities face issues associated with lymphocyte activation and presentation of antigenic fragments to the immune system.

## Conclusions

Grass carp is one of the most extensively farmed fish species in China as it shows fast growth and strong disease resistance. Using Illumina RNA-seq and SMRT sequencing, we herein noted that high stocking density distorted lipid and carbohydrate metabolism in grass carp and also affected their immunocompetence by affecting the expression levels of genes encoding MHC molecules. Our findings emphasize the benefits and importance of maintaining reasonable density in grass carp aquaculture and provide a foundation for further studies.

## Materials and methods

### Fish materials and RNA preparation

Grass carp juveniles (n = 400, 31.3 ± 7.3 g), obtained from the Center of Grass Carp Breeding (Jiangsu, China), were randomly distributed and acclimated in recirculating water tanks for 14 days. A net was implemented in the tank to regulate farming densities, and the fish were randomly divided into two groups: high density (HD) at 40 kg m^− 3^ (mimicking intensive farming density) and low density (LD) at 3 kg m^− 3^ (extensive farming density). The fish were fed three times each day for a month (2 % of total biomass) before tissue collection. Three individuals per group were randomly sampled and anesthetized by hypothermia. Nine different tissues, including the muscle, gill, kidney, heart, spleen, intestine, liver, fin, and brain, were immediately frozen in liquid nitrogen for total RNA extraction, which was achieved using TRIzol (Invitrogen, USA). RNA quality was assessed on 1 % agarose gels, RNA concentration and integrity were examined using an Agilent 2100 Bioanalyzer (Agilent Technologies, USA).

### 4.2 Single-Molecule Real-time Sequencing

#### SMRT library construction and sequencing

Total RNA samples from nine different tissue samples obtained from the LD group were pooled in equal quantities to construct libraries. RNA samples were enriched by oligo(dT) and reverse transcribed using the Clontech SMARTer PCR cDNA Synthesis Kit (USA). Large-scale PCR amplification was performed to generate barcode full-length cDNA. The Blue Pippin Size Selection System (Pacific Biosciences) was used to select > 4-kb fragments of cDNAs, and after enrichment, they were amplified by PCR and then mixed with unscreened cDNA at the same molar mass. After purification and end repairing, full-length cDNA was ligated to SMRT dumbbell adapters. The cDNA products were then purified for Iso-Seq SMRTbell library construction, and after library examination, they were sequenced on the PacBio Sequel platform. Sequencing was performed by Novogene Co. (Beijing, China).

#### PacBio reads processing and full-length transcript analysis

The raw data from the PacBio Sequel platform was processed using SMRT links 5.0 (min Length = 200, min-Read-Score = 0.65) to obtain effective subreads. Circular consensus sequences (CCSs) were then filtered from subreads (min Passes = 2, min Predicted Accuracy = 0.8). Depending on whether 5ʹ- and 3ʹ-cDNA primers were present and whether there was a poly-A tail signal preceding the 3ʹ-primer, a CCS or subread sequence was divided into full-length, non-full length, and chimeric reads. Iterative Clustering for Error Correction was used to obtain consensus reads. Polished consensus reads were acquired from the original consensus corrected with non-full-length reads using the Arrow software. These polished consensus reads were counted and then used for further analysis.

#### Correction of polished consensus sequences and function annotation

To improve accuracy, we used Illumina RNA-seq data to correct polished consensus sequences with LoRDEC [53]. A Python script (TAPIS) was used to further rectify and cluster polished consensus sequences to remove redundancy [54]. Non-redundant genes were compared to the reference genome with the Genomic Mapping and Alignment Program (minimum interval length = 48 nt) [55]. New transcripts were defined as reads that did not match to the reference genome GTF file. To obtain comprehensive annotation information, functions of new transcripts were annotated by searching the Nr (non-redundant protein sequences), Nt (non-redundant nucleotide sequences), Pfam (Protein family), KOG (euKaryotic Ortholog Groups of proteins), Swiss-Prot (A manually annotated and reviewed protein sequence database), KEGG (Kyoto Encyclopedia of Genes and Genomes), and GO (gene ontology) databases.

### Gene structure analysis

High quality transcripts obtained from polished consensus sequences were used to analyze transcription characteristics, including AS events, APA sites, and lncRNAs. AS and APA analyses were performed using the Python package SUPPA2 [54, 56], and used the R package ggplot2 and VennDiagram for drawing. Coding potential prediction of lncRNAs was performed using PLEK, CNCI, and CPC and the Pfam database [57].

### Illumina sequencing

#### Illumina library construction and sequencing

Illumina sequencing was performed using three tissues (the intestine, brain, and muscle) obtained from three fish in each group. RNA was extracted as previously described and enriched by oligo(dT) beads. Fragmentation was carried out using divalent cations under elevated temperature in NEBNext First Strand Synthesis Reaction Buffer (5×). First strand cDNA was synthesized by reverse transcriptase; RNA was then digested by RNaseH, and second strand cDNA was synthesized by DNA polymerase I system. After purification and repair, poly(A) was added to double-stranded cDNA and sequencing adapters were connected. AMPure XP beads were used to screen cDNA (250–300 bp) for PCR augmentation. After secondary purification, the library was constructed and quantified by qRT-PCR (≥ 2 nM), followed by sequencing on Illumina HiSeq 4000. Sequencing was performed by Novogene Co. (Beijing, China). Clean reads (> 100 bp) were obtained by removing reads containing adapters or poly-Ns from the raw data. Clean reads were mapped to the reference genome of *C. idella* with HISAT2 [58].

#### Differential expression gene structural analysis, functional annotation and enrichment

To explore the expression patterns of genes, mean fragments per kilobase million (FPKM) > 00.1 and coefficient of variation of FPKM (standard deviation / mean) > 2 were used as the threshold to filter transcripts for hierarchical and k-means clustering analysis in R. Transcripts with significantly differential expression were identified as those with fold change of FPKM2 > 2 and FDR-adjusted *P* < 0.05 using the HTSeq command between pairwise samples [59]. Fold changes were calculated with the LD group as the reference. To identify the characteristics of DEGs, we matched their gene IDs to the SMRT sequencing data to detect AS events, APA sites, and lncRNAs. DEG structure analyses were performed before annotation. Functional annotation of sequences was achieved using the reference genome and SMRT sequencing data. For clustering analysis, heatmap plots were constructed based on log10 transformed relative intensities of DEGs between the groups. The selection criterion for enriched KEGG pathways and GO terms was set as *P* < 0.05, and used the R package ggplot2 for draw histograms of KEGG pathways and GO terms.

#### Real-time fluorescence quantitative PCR verification of DEGs

First strand cDNA was synthesized from total RNA (1 µg) using reverse transcriptase. The product was then diluted to 200 µL before RT-PCR. Twelve primer pairs were designed using Primer Premier v.5.0 (Table S11) based on DEGs from the second-generation sequencing analysis. PCR fragments were quality tested on 1 % agarose gel and sequenced using the Sanger method. qRT-PCR was performed on a Bio-Rad system using SYBR. Each reaction cycle included initial denaturation at 95 °C for 10 s, followed by 40 cycles of 95 °C for 15 s, 60 °C for 30 s, and 72 °C for 30 s with fluorescence detection. Relative gene expression levels were assessed using the 2^−△△CT^ method, and 18 s rRNA used as the internal standard [60]. We used Independent-Samples T Test (SPSS) to analyze gene expression levels between the LD and HD groups, and used SigmapPlot 14.0 to draw histogram. *P* < 0.05 indicated statistical significance.

## Supplementary Information

**Additional file 1:** Validation RNA-seq profiles by qRT-PCR**. **Data were expressed as mean±standard deviation. The significance of differences between LD and HD groups was analyzed by t’test (*P*< 0.001, ***). Abbreviations: *amy2a*, pancreatic alpha-amylase-like; *cpa4*, carboxypeptidase A4; *vil1*, villin 1; *tm4sf4*, transmembrane 4 L six family member 4;*slc4a1*, solute carrier family 4, anion exchanger, member 1a; *alas2*, aminolevulinate, delta-, synthase 2; *cd99l2*, CD99 molecule-like 2; *bhmt*, betaine-homocysteine methyltransferase; *myom2b*, myomesin 2b; *ubap2b*, ubiquitin associated protein 2b; *grp78*, glucose-regulated protein 78; *sqstm1*, sequestosome 1; *txnipa*, thioredoxin interacting protein a.

**Additional file 2:** The gene structure optimization information by PacBio SMRT sequencing.

**Additional file 3:** Detail information of APA, AS, and lncRNA.

**Additional file 4:** Detail information of APA, AS, and lncRNA.

**Additional file 5:** Detail information of APA, AS, and lncRNA.

**Additional file 6:** The up-regulated and down-regulated DEGs in three tissues of grass carp (FDR ≤ 0.05 & FC ≥ 2).

**Additional file 7:** DEGs simultaneously dysregulated in three tissue.

**Additional file 8:** DEGs related to Gene Ontology enrichment analysis.

**Additional file 9:** DEGs related to KEGG analysis.

**Additional file 10:** DEGs enriched in cholesterol metabolism pathway.

**Additional file 11:** DEGs enriched in carbohydrate digestion and absorption.

**Additional file 12:** Primer sequences of selected genes for quantification of expressions by qRT-PCR.

## Data Availability

All the data supporting our findings are contained within the manuscript. All raw transcriptome data reported in this article have been deposited in the Sequence Read Archive (SRA) under accession number SRP228527.

## Abbreviations

SMRT: Single-Molecule Real-Time
DEGs: Differential expressed genes
AS: Alternative splicing
APA: Alternative polyadenylation
LncRNAs: Long noncoding RNAs
TF: Transcription Factor
CCS: Circular consensus sequences
PASs: Polyadenylation sites
HD: High density
LD: Low density
FPKM: Fragments per kilobase million
GO: Gene Ontology
KEGG: Kyoto Encyclopedia of Genes and Genomes
qRT-PCR: Real-Time Quantitative Reverse Transcription PCR
