# Supplementary figures and images for: Transcriptomic analysis to elucidate the effects of high stocking density on grass carp (Ctenopharyngodon idella)
Source: BMC Genomics. 2021 Aug 16;22:620. doi: 10.1186/s12864-021-07924-4 (PMC8369720; doi:10.1186/s12864-021-07924-4)

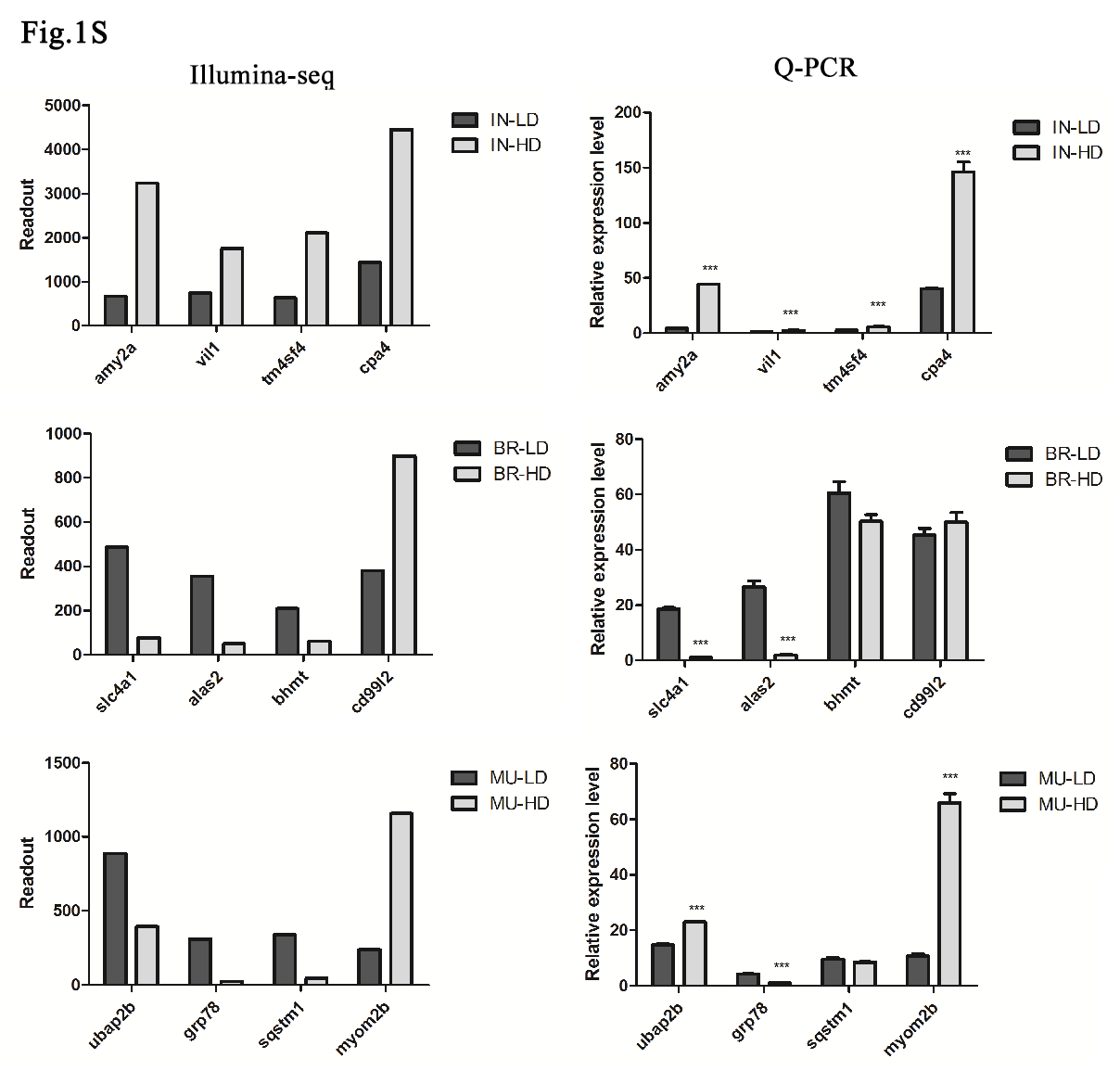

Supplement: Supplementary file 1 — Additional file 1: Validation RNA-seq profiles by qRT-PCR. Data were expressed as mean±standard deviation. The significance of differences between LD and HD groups was analyzed by t’test (P< 0.001, ***). Abbreviations: amy2a, pancreatic alpha-amylase-like; cpa4, carboxypeptidase A4; vil1, villin 1; tm4sf4, transmembrane 4 L six family member 4;slc4a1, solute carrier family 4, anion exchanger, member 1a; alas2, aminolevulinate, delta-, synthase 2; cd99l2, CD99 molecule-like 2; bhmt, betaine-homocysteine methyltransferase; myom2b, myomesin 2b; ubap2b, ubiquitin associated protein 2b; grp78, glucose-regulated protein 78; sqstm1, sequestosome 1; txnipa, thioredoxin interacting protein a. [file 12864_2021_7924_MOESM1_ESM.tif]
